# Personalized Approach for Obese Patients Undergoing Endoscopic Sleeve Gastroplasty

**DOI:** 10.3390/jpm11121298

**Published:** 2021-12-04

**Authors:** Maria Valeria Matteo, Marika D’Oria, Vincenzo Bove, Giorgio Carlino, Valerio Pontecorvi, Marco Raffaelli, Daniela Chieffo, Alfredo Cesario, Giovanni Scambia, Guido Costamagna, Ivo Boškoski

**Affiliations:** 1 Digestive Endoscopy Unit, Fondazione Policlinico Universitario Agostino Gemelli IRCCS, 00168 Roma, Italy; mariavaleria.matteo01@icatt.it (M.V.M.); vincenzo.bove@policlinicogemelli.it (V.B.); giorgio.carlino02@icatt.it (G.C.); valerio.pontecorvi@unicatt.it (V.P.); guido.costamagna@unicatt.it (G.C.); 2 Centre for Endoscopic Research Therapeutics and Training (CERTT), Università Cattolica del Sacro Cuore, 00168 Rome, Italy; 3 Scientific Directorate, Fondazione Policlinico Universitario Agostino Gemelli IRCCS, 00168 Rome, Italy; marika.doria@policlinicogemelli.it (M.D.); alfredo.cesario@policlinicogemelli.it (A.C.); giovanni.scambia@policlinicogemelli.it (G.S.); 4 Endocrine and Metabolic Surgery Unit, Fondazione Policlinico Universitario Agostino Gemelli IRCCS, 00168 Rome, Italy; marco.raffaelli@unicatt.it; 5 Department of Life Sciences and Public Health, Faculty of Medicine and Surgery, Università Cattolica del Sacro Cuore, 00168 Rome, Italy; danielapiarosaria.chieffo@policlinicogemelli.it

**Keywords:** obesity, bariatric endoscopy, endoscopic sleeve gastroplasty, personalized treatment, multidisciplinary team

## Abstract

Obesity is a chronic, relapsing disease representing a major global health problem in the 21st century. Several etiologic factors are involved in its pathogenesis, including a Western hypercaloric diet, sedentariness, metabolic imbalances, genetics, and gut microbiota modification. Lifestyle modifications and drugs often fail to obtain an adequate and sustained weight loss. To date, bariatric surgery (BS) is the most effective treatment, but only about 1% of eligible patients undergo BS, partly because of its negligible morbidity and mortality. Endoscopic sleeve gastroplasty (ESG) is a minimally invasive, endoscopic, bariatric procedure, which proved to be safe and effective. In this review, we aim to examine evidence supporting the role of a personalized and multidisciplinary approach, guided by a multidisciplinary team (MDT), for obese patients undergoing ESG, from patient selection to long-term follow-up. The cooperation of different health professionals, including an endocrinologist and/or obesity medicine physician, a bariatric surgeon, an endoscopist experienced in bariatrics, a registered dietitian, an exercise specialist, a behaviour coach, a psychologist, and a nurse or physician extender, aims to induce radical and sustained lifestyle changes. We also discussed the relationship between gut microbiota and outcomes after bariatric procedures, speculating that the characterization of gut microbiota before and after ESG may help develop new tools, including probiotics, to optimize weight loss outcomes.

## 1. Introduction

Obesity is a major global health problem of the 21st century, affecting 650 million adults worldwide, with 2 billion additional overweight adults [1]. Obesity is a chronic, relapsing disease, classified as a body mass index (BMI) of 30 kg/m^2^ or above [1], and characterized by excessive and aberrant body fat accumulation resulting from the interaction between several etiologic factors, among which are the excessive caloric intake typical of Western diet, sedentariness, incorrect sleep habits, metabolic disorders, gut microbiota, and genetics [2,3]. 

Alterations in the gut microbiome were recently associated with the pathogenesis of obesity and metabolic diseases because of impairments in the regulation of energy balance, adipose deposition, and insulin resistance, along with central appetite regulation [3,4]. Obesity can be associated with a reduction in bacterial diversity and alterations in its composition, such as a decreased abundance of Bacteroidetes, Verrucomicrobia and Faecalibacterium prausnitzii, as well as an increased abundance of Firmicutes phylum and Actinobacteria phylum [3].

Non-invasive approaches to obesity, including diet, physical exercise and drugs, often fail to induce an adequate and prolonged weight loss [5]. Although bariatric surgery is the most effective treatment for morbid obesity, only about 1% of patients undergo surgery because of its non-negligible morbidity and mortality and absent or incorrect information [5,6]. In recent years, the need for bridging the therapeutic gap between conservative approaches and bariatric surgery led to the development of endoscopic bariatric procedures to provide minimally invasive, and more accessible and attractive therapeutic options for patients with obesity who fail non-interventional treatments [7].

Endoscopic sleeve gastroplasty (ESG) is a restrictive transoral procedure that aims to reduce gastric volume and modify gastric motility by placing full-thickness sutures along the greater curvature of the gastric body, thus giving the stomach a sleeve-like conformation. The procedure induces an early satiation, prolonged satiety and delayed gastric emptying resulting in weight loss [8].

Given the complexity and chronic relapsing nature of obesity, a multidisciplinary and personalized approach guided by a bariatric multidisciplinary team seems to be crucial for an adequate and sustained weight loss after a bariatric procedure, whether it is surgical or endoscopic [9,10]. According to the American Society for Gastrointestinal Endoscopy (ASGE), the multidisciplinary team should include an endocrinologist and/or obesity medicine physician, a bariatric surgeon, an endoscopist experienced in bariatrics, an anaesthesiologist, a registered dietitian, an exercise specialist, a behaviour coach, a psychologist, and a nurse or physician extender that coordinates the team [11,12].

In this review, we aim to investigate the impact of a personalized and multidisciplinary approach on the outcomes of ESG with the stapling devices used in clinical practice, and to evaluate the eventual impact of the gut microbiota in this regard. 

## 2. Materials and Methods

An extensive review of the literature on bariatric endoscopic interventions using stapling devices was performed using the MEDLINE (PubMed) database up to 28th October 2021 as reported in Table 1. 

We chose all clinical studies reporting weight loss outcomes of endoscopic sleeve gastroplasty (ESG) performed with Apollo Overstitch, Endomina, Incisionless Operating Platform™ (IOP) for Primary Obesity Surgery Endoluminal (POSE) 2-0, with no limitations on study design. An additional search among the references of the included studies was also carried out to detect for additional relevant studies. The quality appraisal was completed according to the exclusion criteria reported in Table 1. For each study selected, we evaluated the efficacy outcomes, the presence of a multidisciplinary program and the type of health care workers involved in the program. The results of our research were synthesized in Table 2 and narratively discussed.

## 3. Results

To date, several stapling devices were introduced in clinical practice to perform an ESG, namely Apollo Overstitch, Endomina, the incisionless operating platform (IOP) for POSE 2-0, and a new automated device (EndoZip TM) that is under investigation. In our review, we included 21 studies that evaluated endoscopic sleeve gastroplasty, sixteen of which were performed with Apollo Overstitich, three with Endomina, and two with IOP for POSE-2. The total number of patients, the device employed for ESG, the weight loss outcomes, and the post-procedural ancillary programs for each study are summarized in Table 2.

Indication to ESG was established for obese patients (Body Mass Index—BMI ≥ 30 kg/m^2^) who failed previous attempts with diets and lifestyle modifications after the exclusion of contraindications to ESG, including prior gastric surgery, neoplastic or bleeding gastric lesions, coagulopathy, pregnancy, and psychiatric disorders. The pre-procedural evaluation process by the multidisciplinary team and the kind of specialists involved were not fully explained by all the studies. Three ESG studies [17,19,22] did not report any information about the health professionals involved in the pre-operative phase, although, in one study, the authors specified that ESG should be performed in the setting of a multidisciplinary weight management program [17]. The remaining studies mention the pre-procedural involvement of at least the nutritionist and of the psychologist/psychiatrist.

The post-procedural follow-up programs are heterogenous among the studies in terms of the professionals involved, timing and obligatoriness. We considered, as a “multidisciplinary ancillary follow-up program”, the presence of at least nutritional and psychological, scheduled, post-procedural contacts, as well as the follow-up visits with the endoscopist. 

### 3.1. Apollo Overstitch

Of the sixteen studies evaluating the outcomes of ESG with Apollo Overstitch, seven included a multidisciplinary program and nine included only nutritional follow-up or did not explicate the presence of an ancillary follow-up program. 

Since 2014, Lopez Nava et al. have published four studies evaluating ESG with Apollo, with an increasing number of patients and longer follow-up overtime [13,15,16,20]. The authors specify in each paper that all patients were included in a well-structured multidisciplinary program, foreseeing nutritional and psychological consults (weekly or biweekly) and a physical activity program supervised by an exercise physiologist. Interestingly, in their early series including 25 patients, the number of nutritional and psychological contacts (face-to-face, telephone call, emails) could predict weight loss parameters, with patients with high adherence to nutritional and psychological follow-up showing a significantly higher mean percentage of TBWL (>20%) and EWL (>70%) at one year than those with a low adherence (TBWL < 15%, EWL < 50%) [16]. 

In two subsequent studies with similar multidisciplinary follow-up programs, Sartoretto et al. [22] and Barrichello et al. [25] reported a %TBWL of 14.9% and 14.25% at 6 months, respectively. Barrichello and colleagues also showed a %TBWL of 15.06% at 1 year. More recently, Bhandari et al. described a %TBWL of 19.9% at 1 year after ESG in 53 Indian patients who were closely followed-up by a team composed of nutritionists, psychologists, and endocrinologists. Interestingly, the loss to follow-up was minimal [26].

Three studies did not provide information about the presence and the typology of health professionals involved in the follow-up. Abu Dayyeh et al. showed an excess body weight loss (EWL) of 53 ± 17%, 54 ± 40%, and 45 ± 41% at 6, 12, and 20 months in 25 patients treated with ESG, while TBWL was not reported. A standardized healthy lifestyle modification program was encouraged, but no surveillance was carried out during the study in [19]. In a cohort of 77 patients, Kumar et al. described mean TBWL of 16.0% and 17.4% at 6 and 12 months, respectively, though the authors did not provide details on dietary and lifestyle counselling [17]. A large multicentre study of 248 patients reported %TBWL of 15.2% and 18.6% at 6 and 24 months, respectively, with similar results between centres [21]. Weight loss at 6 months was a predictor of weight maintenance along with weight loss at 2 years, suggesting that a %TBWL lower that 10% at 6 months could be an early predictor of long-term failure [21]. 

Instead, the following studies foresaw an ancillary nutritional follow-up, as well as the visits with the endoscopist, and no other specialist consults were mentioned.

In a prospective study by Sharaiha et al., including 91 patients, the mean TBWL was 17.6% at 12 months (76% follow-up), and 20.9% at 24 months (66% follow-up) after the ESG. This study also showed a significant improvement in obesity-related comorbidities, in terms of reduction in levels of haemoglobin A1C, systolic blood pressure, alanine aminotransferase and serum triglycerides [18]. A previous experiment by the same authors showed a %EWL of 30% at 6 months in 10 patients [14].

Graus-Morales at al. reported a mean TBWL of 18.18% (148 patients) at 12 months and 15.53% (72 patients) at 18 months after ESG [23]. 

In the largest series published so far, Alqahtani et al. reported a TBWL of 13.7% at 6 months (369 patients), of 15% at 12 months (216 patients), and of 14.8% at 18 months (*n* = 54). This study also showed the significant impact of ESG on comorbidities, in terms of remission of hypertension (*n* = 28/28 patients), dyslipidaemia (*n* = 18/32 patients) and diabetes (13/17 patients) [24]. 

In a recent, prospective study including 118 obese patients with non-alcoholic fatty liver diseases undergoing ESG, Hajifathalian et al. reported a TBWL of 15.5% (*n* = 78) at 2 years after ESG as well as a significant and sustained improvement in the estimated hepatic steatosis and fibrosis [28]. 

Long-term data are available in a prospective study by Sharaiha et al. including a cohort of 216 patients, 203, 96, and 38 of whom completed a follow-up of 1, 3 and 5 years post-ESG, respectively. The mean %TBWL described was 15.6%, 14.9% and 15.9% at 1, 3 and 5 years, respectively. Compliance with the scheduled visits (with endoscopists and nutritionists) was a predictor of higher TBWL during follow-up [27].

The mean percentage of TBWL in the studies with a multidisciplinary ancillary program was 16% at 6 months, 18.2% at 12 months, and 19.5% at 18–24 months, while in the studies with only nutritional or non-scheduled ancillary follow-up %TBWL was 15.1%, 16.6% and 17% at 12 and 18–24 months, on average (Table 3). The mean percentages of excess weight loss (EWL) are 52.7% and 53.3% at 6 months, 55.9% and 61.8% at 12 months, and 59% and 57.6% at 18–24 months in studies with and without a multidisciplinary follow-up, respectively (Table 3).

### 3.2. Endomina and POSE-2 Procedure

In the studies evaluating ESG with Endomina [29,30,31], the patients treated were offered a nutritional ancillary follow-up, while a psychological post-operative support was provided case-by-case (Table 2). With regard to the POSE-2 procedure, the study by Lopez-Nava et al. mainly focused on the technique, and thus no information is available about an eventual ancillary follow-up [32]. In the study by Jirapinyo et al. patients were scheduled for a nutritional follow-up at 45 days from the procedure, and encouraged to participate in a physical activity program, though a structured follow-up program was not mentioned [33]. 

As mentioned above, ESG with Endozip is still under investigation. To date, only one study has been published on this topic, reporting a mean %TBWL of 16.2% at 6 months in 11 patients provided with a multidisciplinary program [34].

None of these studies on endoscopic suturing techniques analyzed the gut microbiota in obese patients before and after the procedure, and, to our knowledge, the impact of gut microbiota on outcomes of endoscopic bariatric procedures remains unexplored.

## 4. Discussion

The multifactorial and chronic-relapsing nature of obesity requires a multidisciplinary and personalized approach built by a bariatric multidisciplinary team [11,35,36]. The team should have an active role both in patient selection and in the follow-up to support weight loss after any interventional bariatric procedure, whether surgical or endoscopic, since the achievement and maintenance of a satisfactory weight loss requires a solid basis of lifestyle modifications, including diet, physical activity, and behavioural changes [10,11]. The choice of the best suitable procedure by the team should be guided by a comprehensive, pre-operative evaluation, including medical history and previous weight loss attempts, physical examination, complete laboratory tests, esophagogastroduodenoscopy with biopsies, nutritional and psychological or psychiatric counselling [9,10,11,37]. 

Among the endoscopic bariatric treatments, ESG was proven to be effective in ensuring weight loss at 1–2 years (Table 2), with initial evidence of long-term efficacy for up to 5 years [27], and in the alleviating of obesity-related comorbidities [18,24,28]. In a recent study, ESG achieved a significantly higher %TBWL than both intragastric balloon insertion (20.6% vs. 13.9%) and high-intensity and lifestyle therapy (20.6% vs. 14.3%), although ESG showed a significantly lower %TBWL when compared with laparoscopic sleeve gastrectomy (LSG) (17.1% vs. 23.6%) [38]. Observational studies [39,40] and a meta-analysis [41] comparing the endoscopic and surgical restrictive approaches (ESG vs. LSG), showed better weight loss outcomes for surgery. However, ESG demonstrated a better safety profile than surgery, with a rate of serious adverse events (SAEs) of 1.1%, and no fatalities reported [6,42]. Though bariatric surgery is the most effective available therapy for morbid obesity, a significant proportion of patients experience weight regain [43,44,45], with the re-emergence of comorbidities and deterioration in health-related quality of life [46,47], because of nutritional non-compliance, physical inactivity, mental health, and metabolic/endocrine imbalances [47]. These limits can be overcome by a post-operative personalized and multidisciplinary support with regular and periodic contacts [13,48]. 

According to literature, the ideal candidate for ESG as a primary therapy is a patient with a BMI of 30–40 kg/m^2^, with or without medical comorbidities, who fails conservative interventions [7,11,36]. However, ESG can be an attractive, minimally invasive strategy in other conditions that preclude surgery [7,11,36], such as superobese patients (BMI ≥ 50 kg/m^2^). These patients have an excessive anaesthesiologic risk or adhesions/giant incisional hernia due to previous surgery; they are liver or kidney transplant candidates excluded from the waiting list due to BMI ≥ 35 kg/m^2^, in order to improve their transplant candidacy [49,50,51,52,53,54]. Since endoscopic suturing is repeatable, class III obese or superobese patients who refuse or have surgical contraindications, may be candidates for a two-step endoscopic approach, with a second ESG performed after 6–12 months to improve weight loss results with a less invasive and safer approach [55]. 

**Figure 1:**
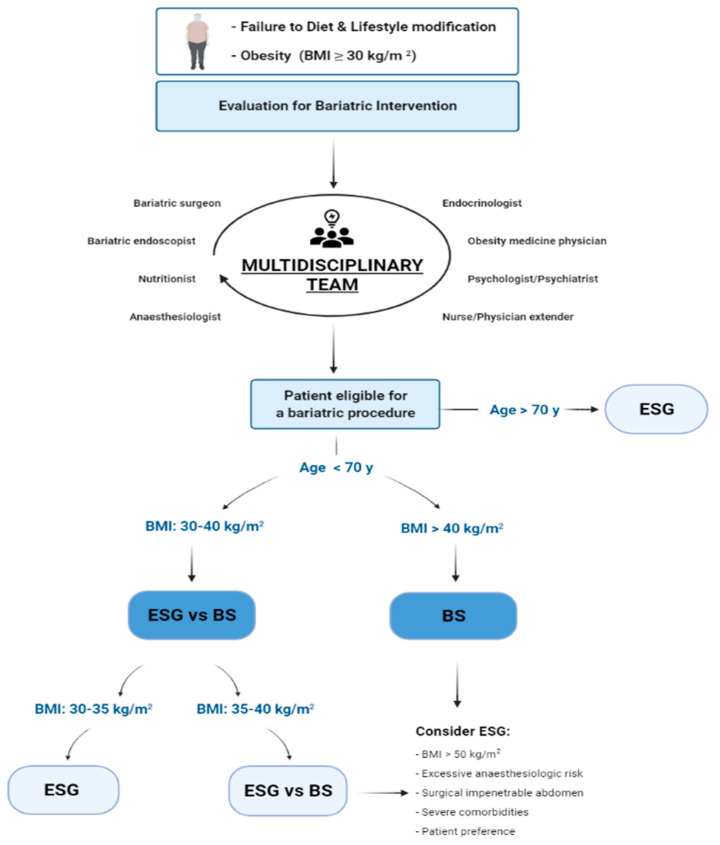
Flow chart for patient selection. BMI: Body Mass Index, BS: Bariatric Surgery, ESG: Endoscopic Sleeve gastroplasty.

A multidisciplinary and personalized program is even more relevant after an ESG that is less efficacious than surgically restrictive interventions since it does not induce irreversible anatomical changes and the opening of the sutures may occur, especially in the case of the inappropriate eating behaviour of the patient [41]. Correct patient selection for a bariatric procedure is complex and requires multi-professional interactions (Figure 1).

To date, the impact of the multidisciplinary team on ESG outcomes has been poorly investigated and the post-procedural monitoring programs are heterogenous among the studies in terms of the professionals involved, timing, and obligatoriness (Table 2). A nutritional follow-up is almost always scheduled, unlike the psychologist/psychiatric or other specialist follow-ups. The mean rates of TBWL obtained by averaging the TBWL of the studies in the multidisciplinary ancillary program, including at least nutritional and psychological follow-up, are higher compared with those with only nutritional or non-scheduled ancillary follow-up (Table 3).

Lopez-Nava demonstrated that a high compliance with the follow-up after ESG is associated with a higher weight loss at 1 year. The same group recently published another study including 962 patients, where half of the patients were treated with intragastric balloon placement (Orbera; ReShape Duo), and the other half were treated with endoscopic gastroplasty (Apollo Overstitch or POSE) [56]. The authors showed that patients with a high adherence to multidisciplinary follow-up, including nutritionists, psychologists, and physiotherapists, achieved a significantly higher weight loss independent of the procedure performed, thus suggesting the importance of a solid, comprehensive, bariatric program to ensure satisfactory post-procedure outcomes.

The American Society for Gastrointestinal Endoscopy (ASGE) recommends enrolling patients after an ESG in a long-term care program delivered by a multidisciplinary team for weight loss maintenance [10,11]. Adherence to follow-up must be particularly encouraged. With the COVID-19 pandemic limiting face-to-face visits, the use of remote contacts may be precious in guiding patients on their path to a sustained weight loss. However, non-adherence and loss to follow-up after a bariatric procedure remains an unresolved issue, so patients should be educated on its relevance [16]. The approach of the multidisciplinary team to the patient should be tailored and personalized to maximize individual outcomes. Some authors analyzed predictors of long-term success after ESG, which can be precious in the personalized management of the patient. Sharaiha et al. [18,27] and Barrichello et al. [25] reported that younger age is a predictor for successful weight loss, maybe because of the high social impact of weight excess among young people and of the greater ability of younger people to modify eating and behavioural habits. Older people may therefore need a closer and more dedicated post-operative follow-up. Furthermore, early weight loss, evaluated at 1 [27] and 6 months [22,25], could predict long-term weight loss, indicating that patients with early poor results will likely experience long-term failure without additional tailored treatments. Not all obese patients have the same response to bariatric interventions, including ESG. The identification of predictive factors of success can be extremely useful in building a personalized multidisciplinary care program. The frequent post-procedural interaction between the patients and the multidisciplinary team may allow the early identification of those “at risk of failure” and to promptly modify their care strategy, including the optimization of non-interventional therapy (i.e., a new dietary regimen, physical exercise program, psychoeducational program), and the evaluation of further interventional procedures. 

The role of the gut microbiome in influencing the outcomes of bariatric procedures is an interesting topic of research in the field of personalized medicine. The identification of pre- and post-procedural gut microbial profiles associated with higher weight loss outcomes could help to develop ancillary therapeutic tools, for instance specific probiotics, that may improve ESG weight loss outcomes by favourably modulating gut microbiota. 

Gut microbiome modifications were recently observed after bariatric surgery, with a shifting to more diverse and healthier microbial profiles, and corrections in the abundance of four major phyla, Firmicutes, Proteobacteria, Bacteroidetes and Verrucomicrobia, that seem to have a direct impact on the reduction in adiposity after BS [57,58,59,60]. However, the ways in which pre-operative microbiome composition can predict clinical outcomes of bariatric surgery is still poorly understood [61]. To our knowledge, no studies evaluating the relationship between endoscopic suturing techniques and gut microbiota have been published. This field of research is very interesting since it can provide precious information to build a personalized care process. Identifying the microbiome composition of the best candidate for a bariatric procedure may help patient selection, and the modification of microbial composition in those patients with unfavourable microbial profiles; probiotic/prebiotic or fecal microbiota transplantation (FMT), for instance, could optimize the clinical outcomes of bariatric procedures. Moreover, mapping the gut microbiota before and a few months after ESG (e.g., 6, 12, and 24 months) may identify some changes and if these changes are similar among all the patients, or if patients with different weight losses after ESG show different gut microbial profiles. These data may allow the identification of the gut microbiome profiles associated with better outcomes after ESG and the development methods, including prebiotic/probiotic, to favourably modulate microbiota to improve weight loss.

In summary, ESG is a minimally invasive procedure that proved to be safe and effective in inducing weight loss, especially for patients with mild-to-moderate obesity, though with inferior efficacy when compared with BS. ESG cannot prescind from a long-term follow-up care managed by a bariatric multidisciplinary team, who aim to induce radical and sustained lifestyle changes, including dietary habits, physical activity, and educational compliance, to achieve a satisfactory and long-term weight loss. Each obesity specialist must collaborate for optimal patient care, from initial weight loss to weight loss maintenance. Nonetheless, future research could investigate the comprehensive impact of personalized follow-up on ESG outcomes, as well as the role of gut microbiota and its modulation.

## Figures and Tables

**Table 1:** Research methodology.

Search engine	PubMed
Date	28/10/2021
Query	“Endoscopic sleeve gastroplasty” OR “endoscopic gastric reduction” or “endoscopic gastric plication” or “endosleeve” or “apollo overstitch” or “endomina” or “primary obesity surgery”
Field	Title/Abstract
Text availability	Full text
Publication date	10 years (2011–2021)
Language	English
Article type	Original articles; clinical studies; randomized controlled-trial.
Total results	209
Exclusion criteria	▪ Articles not matching with the topic (e.g., gastroesophageal disease; endoscopic revision of bariatric surgery; re-suturing; training; other indications for endoscopic suturing) but indicated by PubMed algorithm; ▪ Erratum; animal studies, reviews, case reports (*n* < 9), editorials, comments, letters, paediatric population.
Article selected	21

**Table 2:** Summary of weight loss outcomes and post-procedural ancillary programs of each study.

Study	Stapling Device	N. of Patients	Post-Procedural Ancillary Program	TBWL 6 Months (%)	EWL 6 Months (%)	TBWL 12 Months (%)	EWL 12 Months (%)	TBWL 24 Months (%)	EWL 24 Months (%)	TBWL 5 Years (%)	EWL 5 Years (%)
Lopez-Nava et al. [13]	Apollo Overstitch	20	Nutritionist follow-up (weekly or biweekly) Psychologic follow-up (weekly or biweekly) Exercise physiologist supervised physical activity	17.8 ± 7.5	53.9 ± 26.3	NA	NA	NA	NA	NA	NA
Sharaiha et al. [14]	Apollo Overstitch	10	NA	NA	30	NA	NA	NA	NA	NA	NA
Lopez-Nava et al. [15]	Apollo Overstitch	50	Nutritionist follow-up (weekly or biweekly) Psychologic follow-up (weekly or biweekly) Exercise physiologist supervised physical activity	17.2 ± 7.5	53.5 ± 26.2	19.0 ± 10.8	57.0 ± 33.9	NA	NA	NA	NA
Lopez-Nava et al. [16]	Apollo Overstitch	25	Nutritionist follow-up (weekly or biweekly) Psychologic follow-up (weekly or biweekly) Exercise physiologist supervised physical activity	17.8 ± 7.5	53.9 ± 24.8	18.7 ± 10.7	54.6 ± 31.9	NA	NA	NA	NA
Kumar et al. [17]	Apollo Overstitch	77	NA	16.2 ± 0.7	NA	17.4 ± 1.1	NA	NA	NA	NA	NA
Sharaiha et al. [18]	Apollo Overstitch	91	Nutritionist follow-up (not mandatory)	14.4	NA	17.6	NA	20.9	NA	NA	NA
Abu Dayyeh et al. [19]	Apollo Overstitch	25	NA	NA	53 ± 17	NA	54 ± 40	NA	45 ± 41 ^†^	NA	NA
Lopez-Nava et al. [20]	Apollo Overstitch	154	Nutritionist follow-up (weekly or biweekly) Psychologic follow-up (weekly or biweekly) Exercise physiologist supervised physical activity	15.8 ± 7.1	47.8 ± 29.4	18.2 ± 10.1	52.6 ± 31.3	19.5 ± 10.5	60.4 ± 31.1	NA	NA
Lopez-Nava et al. [21]	Apollo Overstitch	248	NA	15.2	NA	NA	NA	18.6	NA	NA	NA
Sartoretto et al. [22]	Apollo Overstitch	112	Nutritionist follow-up * Psychologic follow-up * Exercise physiologist supervised physical activity	14.9 ± 6.1	50.3 ± 22.4	NA	NA	NA	NA	NA	NA
Graus-Morales et al. [23]	Apollo Overstitch	148	Nutritionist follow-up (weekly at the beginning, monthly afterwards)	15.1 ± 4.9	66.0 ± 39	18.2 ± 6.8	77.6 ± 42	17.5 ± 7.6 ^‡^	75.4 ± 85 ^‡^	NA	NA
Alqahtani et al. [24]	Apollo Overstitch	1000	Nutritionist follow-up (monthly during the first year, every 3 months afterwards)	13.7 ± 6.8	64.3 ± 56.2	15.0 ± 7.7	67.5 ± 52.3	14.8 ± 8.5 ^‡^	64.7 ± 55.4 ^‡^	NA	NA
Barrichello et al. [25]	Apollo Overstitch	193	Nutritionist follow-up (biweekly or monthly) Psychologic follow-up (at 1, 3 * ^not mandatory^, 6, and 12 months) Physical educator (monthly) * ^not mandatory^	14.2 ± 5.3	56.2 ± 22.9	15.1 ± 5.2	59.4 ± 25.7	NA	NA	NA	NA
Bhandari et al. [26]	Apollo Overstitch	53	Nutritionist follow-up * Psychologic follow-up * Endocrinologic follow-up *	14.3 ± 6.2	NA	19.9 ± 4.9	NA	NA	NA	NA	NA
Sharaiha et al. [27]	Apollo Overstitch	216	Nutritionist follow-up (at 1, 3, 6, 12, 24 months, and yearly afterward)	NA	NA	15.6 (95% CI, 14.1–17.1)	47.9 (95% CI, 42.4–53.3)	14.9 (95% CI, 12.1–17.7) **	45.1 (95% CI, 34.9–55.2) **	15.9 95% CI, 11.7–20.5)	45.3 (95% CI, 32.9–57.7)
Hajifathalian et al. [28]	Apollo Overstitch	118	Nutritionist follow-up (at 1, 3, 6, 12, and 24 months)	14.6 (13.3–15.9)	45.3 (39.9–50.7)	15.6 (13.9–17.4)	47.8 (41.4–54.2)	15.5 (13.3–17.8)	45.5 (38.1–52.8)	NA	NA
Huberty et al. [29]	Endomina	11	Nutritionist follow-up (at 1 month, every 3 months afterwards)	NA	41	NA	NA	NA	NA	NA	NA
Huberty et al. [30]	Endomina	51	Nutritionist follow-up (at 1 month, every 3 months afterwards)	8.0 (SD 5.0)	31.0 (SD 20.0)	7.0 (SD 7.0)	29.0 (SD 28.0)	NA	NA	NA	NA
Huberty et al. [31]	Endomina	71	Nutritionist Psychologic support (case by case) Physical activity encouraged	11.0 (95% CI: 8.9–13.2)	38.6 (95% CI, 31.1–46.0)	11.9 (95% IC, 9.3–14.5)	42.7 (95% CI, 33.1–52.3)	NA	NA	NA	NA
Lopez-Nava et al. [32]	POSE 2.0	73	NA	15.7	NA	NA	NA	NA	NA	NA	NA
Jirapinyo et al. [33]	POSE 2.0	10	Nutritional counselling (at 45 days) Physical activity recommended	15.0 ± 7.1	37.9 ± 20.0	NA	NA	NA	NA	NA	NA
Lopez-Nava et al. [34]	EndoZip	11	Nutritionist (bi-weekly) Psychologist (bi-weekly) Physiotherapist (bi-weekly)	16.2 ± 6.0	46.5 ± 28.6	NA	NA	NA	NA	NA	NA

TBWL: total body weight loss. EWL: excess weight loss. NA: not available. ^†^: 20 months; ^‡^: 18 months; *: timing unknown; **: 3 years.

**Table 3:** Mean TBWL and EWL after ESG with Apollo Overstitch among the studies according to ancillary post-procedure programs.

Weight Loss Outcomes (Mean Value)	TBWL at 6 Months (%)	EWL at 6 Months (%)	TBWL at 12 Months (%)	EWL at 12 Months (%)	TBWL at 18–24 Months (%)	EWL at 18–24 Months (%)
All studies	15.7	54.4	17.3	57.6	17.4	58.2
Studies with only nutritional or non-specified follow-up	15.1	51.7	16.6	59.0	17.0	57.6
Studies with multidisciplinary follow-up	16.0	52.6	18.2	55.9	19.5	60.4

TBWL: total body weight loss. EWL: excess weight loss.

## Data Availability

Not applicable.
